# Geographical distribution of fertility rates in 70 low-income, lower-middle-income, and upper-middle-income countries, 2010–16: a subnational analysis of cross-sectional surveys

**DOI:** 10.1016/S2214-109X(21)00082-6

**Published:** 2021-05-18

**Authors:** Carla Pezzulo, Kristine Nilsen, Alessandra Carioli, Natalia Tejedor-Garavito, Sophie E Hanspal, Theodor Hilber, William H M James, Corrine W Ruktanonchai, Victor Alegana, Alessandro Sorichetta, Adelle S Wigley, Graeme M Hornby, Zoe Matthews, Andrew J Tatem

**Affiliations:** a WorldPop, Geography and Environmental Science, University of Southampton, Southampton, UK; b GeoData, University of Southampton, Southampton, UK; c Division of Social Statistics, and Demography and Centre for Global Health, Population, Poverty and Policy, University of Southampton, Southampton, UK; d Department of Earth Sciences Centre for Development Research, Freie Universität Berlin, Germany; e School of Geography, and Leeds Institute for Data Analytics, University of Leeds, Leeds, UK; f Department of Population Health Sciences, Virginia Tech, Blacksburg, VA, USA; g Population Health Unit, Kenya Medical Research Institute, Wellcome Trust Research Programme, Nairobi, Kenya; h Centre for Tropical Medicine and Global Health, Nuffield Department of Medicine, University of Oxford, John Radcliffe Hospital, Oxford, UK

## Abstract

**Background:**

Understanding subnational variation in age-specific fertility rates (ASFRs) and total fertility rates (TFRs), and geographical clustering of high fertility and its determinants in low-income and middle-income countries, is increasingly needed for geographical targeting and prioritising of policy. We aimed to identify variation in fertility rates, to describe patterns of key selected fertility determinants in areas of high fertility.

**Methods:**

We did a subnational analysis of ASFRs and TFRs from the most recent publicly available and nationally representative cross-sectional Demographic and Health Surveys and Multiple Indicator Cluster Surveys collected between 2010 and 2016 for 70 low-income, lower-middle-income, and upper-middle-income countries, across 932 administrative units. We assessed the degree of global spatial autocorrelation by using Moran's *I* statistic and did a spatial cluster analysis using the Getis-Ord Gi* local statistic to examine the geographical clustering of fertility and key selected fertility determinants. Descriptive analysis was used to investigate the distribution of ASFRs and of selected determinants in each cluster.

**Findings:**

TFR varied from below replacement (2·1 children per women) in 36 of the 932 subnational regions (mainly located in India, Myanmar, Colombia, and Armenia), to rates of 8 and higher in 14 subnational regions, located in sub-Saharan Africa and Afghanistan. Areas with high-fertility clusters were mostly associated with areas of low prevalence of women with secondary or higher education, low use of contraception, and high unmet needs for family planning, although exceptions existed.

**Interpretation:**

Substantial within-country variation in the distribution of fertility rates highlights the need for tailored programmes and strategies in high-fertility cluster areas to increase the use of contraception and access to secondary education, and to reduce unmet need for family planning.

**Funding:**

Wellcome Trust, the UK Foreign, Commonwealth and Development Office, and the Bill & Melinda Gates Foundation.

## Introduction

An awareness of fertility rate is crucial for describing the demographic profile of a country, opportunities for development, change in population size, and for assessing challenges to women's reproductive health.^1^ There are important health and development implications for countries with high-fertility rates (predominantly low-income and middle-income countries); higher birth rates are associated with lower levels of education, higher deprivation, and greater health risks for children and their mothers.^2^ These greater health risks include a greater risk of child mortality for higher-order births and closely spaced births, and a greater risk of maternal mortality at higher parities and at younger and older ages.^2^ Also, an early childbearing onset could have adverse consequences on the health and the survival of mother and child, educational attainment of the mother, and economic opportunities through the demographic dividend.^3^, ^4^ There are also links between fertility and the risk of infectious diseases, such as the relationship between high fertility and the high rate of mother-to-child HIV transmission,^5^ and the maternal and child health implications of the Zika virus.^5^, ^6^

One aim of the UN Sustainable Development Goals (SDGs) is to address issues concerning the reproductive health and wellbeing of women of all ages.^7^ Specifically, there is a focus on achieving universal access to sexual and reproductive health care to prevent unintended pregnancies and reduce adolescent childbearing (SDG 3.7), and, separately, to achieve gender equality and women and girls' empowerment (SDG 5).^7^ Universal and equitable access to high quality sexual and reproductive health services is yet to be achieved, and there are still vulnerable populations that face major barriers to access, such as those who live in remote areas far away from health facilities, do not have access to transportation, cannot afford costs of care, and single and unmarried women.^8^, ^9^ Unmet needs for contraception, defined as the percentage of women who do not want to become pregnant but are not using contraception, tends to be greater in low-income and middle-income countries than in high-income countries, but with high geographical variation.^10^, ^11^

**Research in context**

**Evidence before this study**

Estimates of age-specific fertility rates (ASFRs) and total fertility rates (TFRs) are extensively used in all forms of demographic, development, and health-related analyses. Since the 1990s, national estimates of ASFRs and TFRs have been produced by the UN Population Division, among others, but an increased need has been recognised for estimates at the subnational level to target policy and geographical prioritisation. ASFRs and TFRs at the subnational level can be estimated through nationally representative household surveys and can serve a wide range of analyses, highlighting in-country heterogeneities. Although subnational fertility estimates have been used in previous studies, these have not looked at geographical clustering of TFRs and at patterns of fertility determinants and background characteristics within those clusters across a wide area in low-income, lower-middle-income, and upper-middle-income countries. We searched PubMed, Google Scholar, and Scopus for articles in English related to subnational data assembly, mapping, and analysis of ASFRs in low-income and middle-income countries between Jan 1, 2006, and Dec 1, 2019. We used the search terms ‘subnational mapping’, ‘total fertility rates’, “age-specific fertility rates”, ‘fertility determinants’ and combinations of each, as well as regional and country-specific terms to capture studies covering low-income and middle-income countries.

**Added value of this study**

This study provides a comprehensive descriptive analysis of the geographical heterogeneities in fertility rates within countries, by assembling subnational ASFRs and TFRs from nationally representative household surveys for a total of 932 administrative units in 70 low-income, lower-middle-income, and upper-middle-income countries. This study was the first to assemble such datasets at this scale, and to investigate geographical clustering of fertility and key determinants. Such heterogeneities are masked when examining only national-level data. The study also highlights areas of clustering of high and low fertility which cross country borders, as well as patterns of characteristics and behaviours in each cluster. The analyses explore how fertility clusters relate to fertility determinants, such as the use of contraception, women's secondary education or higher, and unmet needs for family planning. These findings highlight contiguous subnational geographical areas with similar characteristics, indicating that both subnational and national strategies are needed to reduce inequalities in access to contraception and education.

**Implications of all the available evidence**

This study can help inform the allocation of resources for family planning and women's education. The relationship between high-fertility clusters and areas of low use of contraception, unmet needs for family planning, and low rates of women's education emphasise the need for targeting and maximising impact in the context of resource scarcity. Resources should be targeted to areas where levels of unmet need for family planning are high, and where access to contraception and women's access to education are low. Interventions that respond to these subnational variations will enable progress towards the achievement of the UN Sustainable Development Goals.

Global fertility rates have decreased considerably since the 1970s,^12^ yet the rate of decline is not consistent across countries; for example, areas in sub-Saharan Africa and Asia still have high age-specific fertility rates (ASFRs) and total fertility rates (TFRs; in 2010–15, women in Niger had a TFR of 7·6 children and women in Timor-Leste had a TFR of 5·9 children^13^), and the rate of fertility is projected to decline at a slower pace in sub-Saharan Africa than in Asia, Latin America, and the Caribbean.^13^ Official fertility statistics usually rely on national estimates,^14^ which potentially mask underlying heterogeneity in fertility rates within countries. Disaggregating ASFRs and TFRs at subnational level, and identifying heterogeneities in fertility rates within and between countries, is important for planning and implementing safer maternal and newborn health strategies, and for targeting programmes on sexual and reproductive health care in a more effective way. Furthermore, being able to identify areas of high fertility and targeting these areas to improve coverage and access to family planning is important for programmes aiming to achieve universal health-care access. Moreover, analysing hot spots of high fertility is important in identifying areas of greater need for coverage and better access to family planning, and for informing programmes working to achieve universal health-care access. Analysing fertility determinants in small geographical units and how they relate to clusters of high fertility aid the identification of hotspots where coverage lags behind neighbouring areas.

In this study, firstly we aimed to identify statistically significant pockets of relative high fertility (hot spots, also referred to as high-fertility clusters) and relative low fertility (cold spots, or low-fertility clusters), by using local spatial autocorrelation statistics for subnational fertility rates across 70 low-income, lower-middle-income, and upper-middle-income countries as defined by the World Bank^15^ between 2010 and 2016. Secondly, we aimed to identify areas of relative high need for selected determinants of fertility, to uncover geographical variations in the determinants, and how areas most in need relate with pockets of high fertility. Identifying pockets of relatively high fertility helps in determining whether neighbouring areas may also have similar contexts and share similar characteristics.^16^ This study explored the distribution of proximate determinants of fertility (such as the use of contraception),^17^, ^18^ and social determinants of fertility, also called background characteristics, such as level of female education, place of residence, and national economic profile.^19^ Previous studies found that several different factors affect the variation in TFRs and ASFRs, including cultural, geographical, and socioeconomic barriers.^19^, ^20^, ^21^, ^22^, ^23^ Our aim is not to identify the determinants of fertility or understand their influence on fertility, but to describe patterns of selected key determinants within fertility clusters, and to highlight how geographical heterogeneities and clustering in fertility rates can reflect inequalities in these key selected determinants of fertility.

## Methods

### Study design and data sources

We did a subnational analysis of cross-sectional surveys collected between 2010 and 2016 to analyse fertility rates across 70 countries, including 932 administrative units in Africa, Asia, and Latin America. Fertility data from each administrative unit were obtained from the most recent publicly available and nationally representative Demographic and Health Surveys (DHS) and UNICEF Multiple Indicator Cluster Surveys (MICS). DHS and MICS are typically designed using a stratified two-stage probability sampling design, and provide nationally representative surveys for a number of low-income and middle-income countries. When appropriate data were available from both surveys, we used the most recent source. Data were gathered across 70 low-income, lower-middle-income and upper-middle-income countries. Only a subsample of low-income, lower-middle-income, and upper-middle-income countries is included in this work, as the DHS and MICS do not collect data for all countries (appendix pp 1–3). Ethical approval for this study was granted by the University of Southampton, UK, Ethics Committee (24376).

### Measurement of fertility rates

Estimates of ASFRs and TFRs were used to generate subnational ASFRs and TFRs using methods described by the DHS Guide to Statistics.^24^ The ASFR is defined as the number of births occurring during the 3 years preceding the survey per 1000 women of reproductive age classified in one year or five year age groups, while the TFR refers to the average number of livebirths a woman would have if she was subject to the current ASFRs throughout her reproductive years (aged 15–49 years). The TFR is obtained by summing the grouped ASFRs and multiplying by 5 (which corresponds to the length of time, 5 years, used to categorise the ASFR age groups).^24^, ^25^ In this study, teenage fertility rates are defined as the ASFR for women aged 15–19 years. Corresponding subnational area polygons were obtained from the DHS spatial repository,^26^ allowing us to geographically map the distribution of ASFRs and TFRs. These areas typically correspond to administrative level 1 units, or provinces, and we refer to them in this study as subnational areas. We refer to Africa, Asia, and Latin America as regions.

### Spatial cluster analyses

We did spatial cluster analyses for the TFR indicator to test the presence or absence of significant spatial clustering across subnational areas in the dataset and to characterise those clusters in terms of the relationships among neighbouring subnational areas. Here, cluster refers to agglomerations of adjacent subnational areas, which can show, for example, areas of high concentrations of the observed characteristic surrounded by neighbouring areas with high rates (high–high); or, areas of low concentration surrounded by neighbours with low concentrations (low–low).

We first assessed the degree of global spatial autocorrelation, or the degree to which one spatial unit shares similar or different characteristics with its neighbouring spatial unit, by using the Moran's *I* statistic (using n=999 permutations yielding a pseudo p value of 0·001). The global Moran's *I* statistic indicates whether spatial clustering of a characteristic exists across the area, compared with a null hypothesis of a spatially random distribution. In the context of this study, the spatial units used are administrative units and global Moran's *I* values closer to 0 indicate little or no spatial clustering, whereas positive values indicate spatial clustering where neighbouring units tend to have similar values, and negative values indicate that neighbouring units tend to have different values. We considered each of the 932 subnational regions as spatial units in the context of this study, and the values we assessed under spatial cluster analysis were TFR and key selected fertility determinants.

We used the Gi* statistic^27^ based on local Getis-Ord Gi* hot spot analysis to examine where spatial clusters occur on the landscape by identifying areas with statistically significant clustering of relative high values (hot spots) or relative low values (cold spots) in the three regions. This measure indicates the presence or absence of significant spatial clustering, with the null hypothesis being that there is no difference in characteristic between a unit and its spatial neighbours. The local Getis-Ord Gi* hot spot analysis identifies areas where relatively high or low values tend to cluster together, based on the comparison of the indicator values in each location with corresponding values in neighbouring areas. In this study, when discussing local Getis-Ord Gi* analyses, we refer to high clusters when statistically significant clustering of high rates are shown, low clusters when there are significant clustering of low rates, and to non-significant clustering, or no significant clustering, when no statistically significant clustering of characteristics is shown.

Both the spatial autocorrelation analysis (Global Moran's *I*) and the local Getis-Ord Gi* hot spot analysis were done using GeoDa (version 1.12.1.161).^28^ We used the contiguity edges corner method, also referred to as Queen's Case,^29^ as the conceptualisation method to define the spatial relationship between areas (this method returned a significant, and the highest, Moran's *I* for the three regions), applied randomisation (using n=99 999 permutations), and specified a pseudo p value for clusters of less than 0·01 as the cutoff value. In this study, we did a separate spatial cluster analysis for each of the three regions to capture specific characteristics of each region; therefore, high-fertility clusters and low-fertility clusters are relative to each region. Although clusters are not comparable across regions (eg, high-fertility clusters in Africa cannot be directly compared with high-fertility clusters in Latin America and Asia), between-cluster patterns of ASFR can be compared across the regions. Within each cluster, we plotted the distribution of ASFRs and explored the variation of ASFR patterns between high-fertility clusters and low-fertility clusters. We also extracted from the surveys key selected determinants of fertility data (for the same 932 subnational areas from the household surveys), including the percentage of women with unmet needs for family planning (defined as the percentage of women who do not want to become pregnant but are not using contraception), the percentage of women using any contraceptive method, the percentage of women using modern contraception (such as female sterilisation, male sterilisation, oral contraceptive pills, the intrauterine contraceptive device, injectables, or implants, among others),^21^, ^22^ and the percentage of women with a secondary or higher level of education (appendix p 3).^30^ We plotted determinants of fertility data against each fertility cluster and explored their distribution within high-fertility and low-fertility clusters. We also did spatial cluster analysis for the aforementioned key selected fertility determinants in each of the three regions, following the methods described earlier, to visually assess the presence of overlap of high and low clusters of determinants with TFRs. Finally, subnational ASFRs were plotted by country background characteristics such as area of residence (urban or rural), region,^13^ and income level.^15^

We used descriptive statistics to explore the distribution of ASFRs and of selected fertility determinants in each cluster using Stata SE (version 16) and created maps using Esri ArcGIS (version 10.7).

### Role of the funding source

The funder of the study had no role in study design, data collection, data analysis, data interpretation, or writing of the report.

## Results

**Figure 1:**
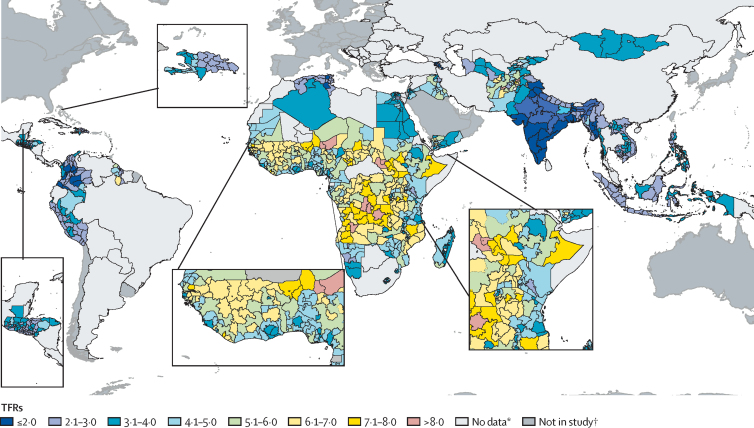
Subnational TFR distribution in Africa, Asia, and Latin America between 2010 and 2016 TFR refers to the number of births per 1000 women of reproductive age occurring during the 3 years preceding the survey. TFR=total fertility rate. *Low-income, lower-middle-income, and upper-middle-income countries where no data were available. †Higher-income countries.

We analysed 70 nationally representative household surveys from the DHS and MICS for the period 2010–16. TFRs were heterogeneous across the three regions (figure 1; appendix p 5). Highest TFRs (between 4 and 8·6) were concentrated mainly in sub-Saharan African countries, with the exception of areas in Kenya, Mozambique, Zambia, Zimbabwe, Namibia, Swaziland, and Lesotho, where TFR was lower than 4. TFRs were lowest (<4) in Latin America, north Africa, and southeast Asia, although some areas in all three regions had TFRs greater than 4. The region with the highest mean TFR was Africa (5·18 children per woman, SD 1·4), ranging from 1·8 in Addis Ababa, Ethiopia, to 8·6 in Bié Province, Angola. In Asia, the mean TFR was 3·41 (SD 1·3), ranging from 1·1 (Armenia) to 8·9 (Nooristan, Afghanistan). Mean TFR in Latin America was 2·88 (SD 0·8), ranging from 1·1 (Caldas, Colombia) to 6·5 (Upper Takutu-Upper Essequibo, Guyana). 36 of the 932 subnational regions studied presented TFRs below 2·1 children per woman (ie, below replacement level). Most of the areas below replacement level were in India, Myanmar, and Armenia in Asia, and Colombia in Latin America. There was substantial variability across countries in the distribution of both TFRs and teenage fertility rates (appendix pp 4–5), and areas of high teenage fertility rates, such as areas of east Africa and Afghanistan, did not appear to always correspond with areas of high TFRs. This observation was supported by the subnational distribution of TFRs and teenage fertility rates in each country within their average spatial resolution groups (appendix pp 1, 2, 7–12). The average spatial resolution measures the effective resolution of administrative units in kilometres (ie, the cell size of administrative units if all units were square of equal size). It is calculated as the square root of the land area divided by the number of administrative units,^31^ and is used for better comparisons between countries.

**Figure 2:**
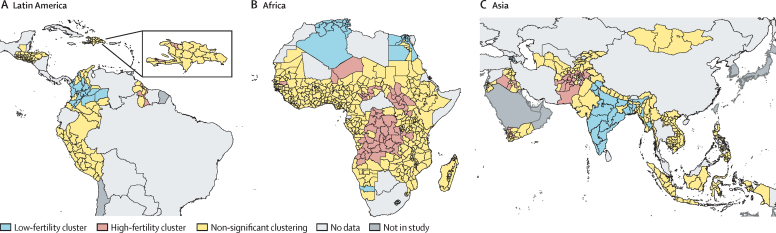
Spatial clusters of fertility in Latin America (A), Africa (B), and Asia (C)

Significant positive spatial autocorrelation of TFRs was observed in all three regions, as measured by the global Moran's *I* (appendix p 12), indicating that neighbouring areas tend to have similar TFR values, forming fertility clusters. Figure 2 shows areas with significant clustering of low–low (cold spots) and high–high (hot spots) fertility in all three regions, as measured through the Getis-Ord Gi* statistic. TFR in high-fertility clusters and low-fertility clusters varied widely according to the region; in Latin America, the TFR in high-fertility clusters was 3·2 children per woman (SD 0·7) and in low-fertility clusters the TFR was 2·1 (0·5). In Africa, the TFR in high-fertility clusters was 7·1 (0·8) and in low-fertility clusters it was 3·1 (0·7). In Asia, the TFR in high-fertility clusters was 5·2 (1·4) and in low-fertility clusters it was 2·0 (0·6; appendix p 6). Clusters of high fertility and low fertility cross national boundaries in some areas of Africa and Asia; areas of high fertility were observed in the bordering countries of Democratic Republic of the Congo, Angola, and Tanzania, as well as in South Sudan, Sudan, and Uganda. Conversely, low-fertility clusters were observed in Algeria and neighbouring Tunisia. In Asia, low-fertility clusters were observed in Myanmar and in India and neighbouring Bangladesh, and a high-fertility cluster was observed in Pakistan and neighbouring Afghanistan.

**Figure 3:**
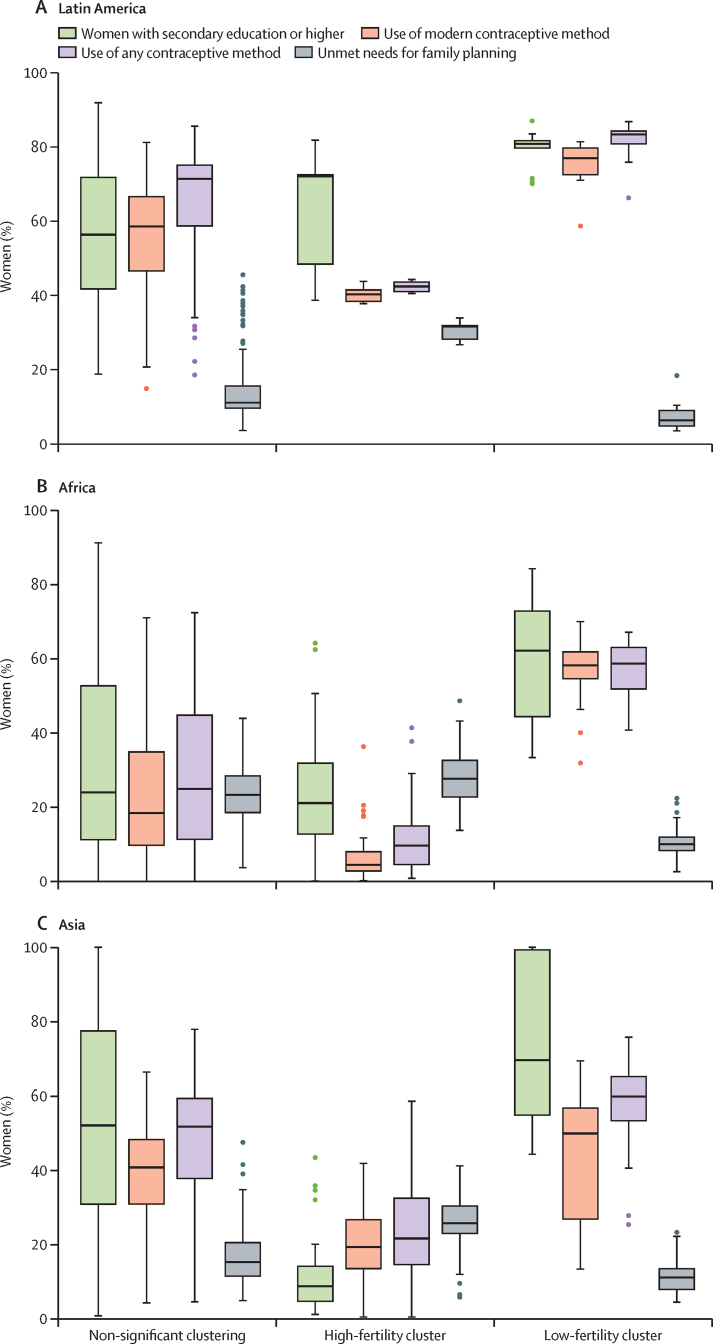
Distribution of key fertility determinants in 932 subnational areas of Latin America (A), Africa (B), and Asia (C) Boxplots showing the minimum (lower whisker), maximum (upper whisker), median (middle line of box), and first and third quartiles (lower and upper ends of box) of the percentage of women with a secondary education or higher, or who use any contraception, or who use modern contraception, or who have unmet needs for family planning. Outliers are plotted as individual points.

**Figure 4:**
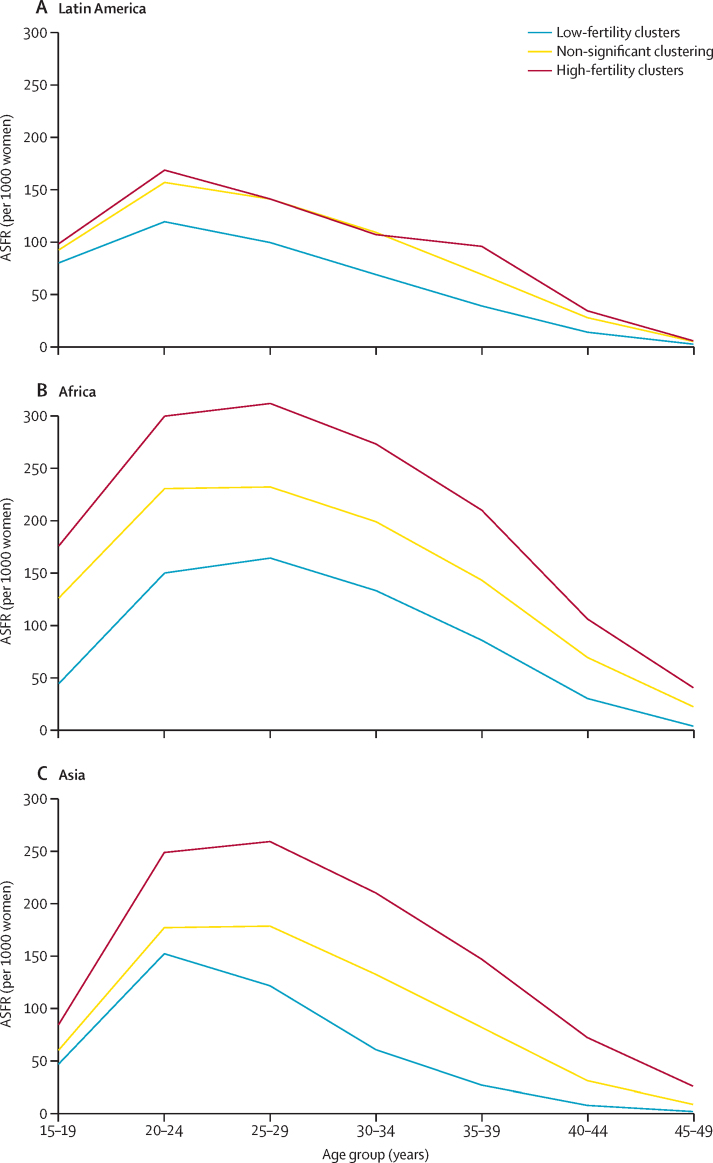
Distribution of ASFRs within fertility clusters in Latin America (A), Africa (B), and Asia (C) The ASFR is defined as the number of births occurring during the 3 years preceding the survey per 1000 women of reproductive age classified in one year or five year age groups. ASFR=age-specific fertility rate.

There are consistent patterns in the distribution of the family planning and education indicators within each cluster in the three regions (figure 3). The percentage of women using contraception of any kind is higher in low-fertility clusters than in high-fertility clusters in all three regions (figure 3). The percentage of women with at least secondary education is higher in low-fertility clusters than in high-fertility clusters across all three regions, and the percentage of women with unmet needs for family planning is higher in high-fertility clusters than in low-fertility clusters (appendix p 8). Comparisons between regions show that, within high-fertility clusters, the median percentages of women using any method of contraception and modern methods of contraception are lowest in Africa (9·7% [IQR 4·3–15·1] and 4·4% [2·6– 8·2], respectively, and highest in Latin America (42·4% [40·8–43·8] and 40·3% [38·2–41·7], respectively). In Africa and Asia, the median percentage of women with secondary education and higher is greater in low-fertility clusters than in high-fertility clusters, with the largest gap between the two clusters shown in Asia. In Asia, the prevalence of women's secondary or higher education is as high as 100% in some regions. There appeared to be a high unmet need for family planning in high-fertility clusters across all regions. In Africa and Asia, ASFRs in high-fertility clusters were consistently higher in every age group than in low-fertility clusters; in Latin America the difference between ASFRs in high-fertility clusters and low-fertility clusters was narrower than in Africa and Asia in almost all age groups (figure 4).

**Figure 5:**
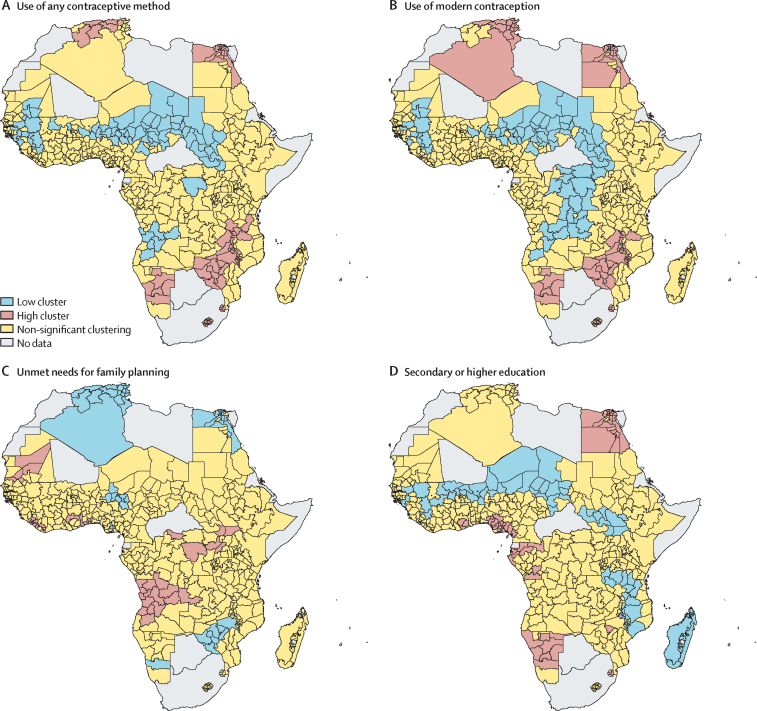
Spatial clusters of high prevalence and low prevalence of key fertility determinants in Africa Fertility determinants were use of any contraceptive method (A), use of modern methods of contraception (B), unmet needs for family planning (C), and having a secondary or higher education (D). Low cluster is an area with significant clustering of low rates of a characteristic. High cluster is an area with significant clustering of high rates of a characteristic.

In Africa, clusters of high prevalence and low prevalence of fertility determinants appeared to be more variable when compared with the fertility clusters. Clusters where the use of any contraception and modern contraception was low appear to have formed in central Africa and southern Africa (west Africa, Nigeria, Niger, Chad, Sudan, South Sudan, Democratic Republic of the Congo, and Angola; figure 5). These areas partially overlapped with high-fertility clusters, and with clusters where the percentage of women with secondary or higher education was low (Figure 2, Figure 5). Low-fertility clusters appeared to show overlap with areas characterised by the high use of contraception (any and modern), high percentage of women with secondary or higher education, and low unmet needs for contraception (Algeria, Tunisia, and Egypt in north Africa; Namibia, Zimbabwe, Malawi, and Tanzania in southern Africa and east Africa). In Latin America, areas where there was a high prevalence of the use of any contraception, the use of modern contraception, women with secondary or higher education, and low unmet need for family planning showed a high degree of overlap with low-fertility clusters (appendix pp 12–13). A similar overlap was seen between clustered areas of low prevalence of these characteristics and high-fertility clusters, with the exception of a cluster of low prevalence of women with secondary or higher education in Guatemala and neighbouring Honduras that corresponded to an area of non-significant fertility clustering (appendix pp 12–13). In Asia, Pakistan and Afghanistan showed a low prevalence of any contraceptive use, modern contraceptive use, and women with secondary or higher education, and high fertility rates (appendix pp 12–13). Low fertility rates in southeast India appeared to correspond with a high prevalence of modern methods of contraception. The high-fertility clusters seen in certain areas of Iraq appeared to correspond to areas of low prevalence of unmet need for family planning, suggesting that fertility patterns were not driven by lack of family planning resources.

**Figure 6:**
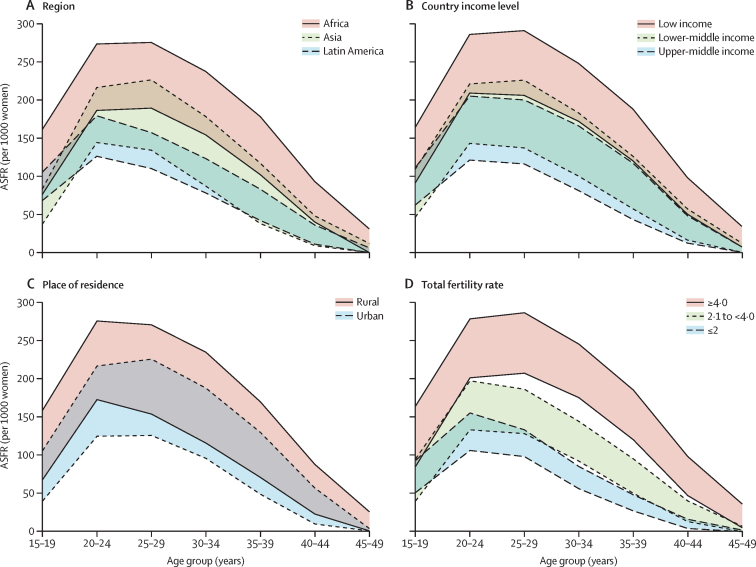
Subnational ASFRs in the areas included in this study by region (A), country income level (B), place of residence (C), and total fertility rate (D) The subnational ASFRs were plotted by country background characteristics, using the subnational upper and lower quintiles. ASFR=age-specific fertility rate.

Subnational analysis of ASFRs by categories of selected country background characteristics showed that Africa had the highest ASFRs across all age groups, and that there was a high degree of variability within the region (figure 6A), followed by Asia and Latin America. The subnational ASFRs were plotted by country background characteristics, by using the subnational upper and lower quintiles. In Latin America, ASFR peaks at ages 20–24 years (median ASFR of 151 per 1000 women [IQR 126–179]) and then slowly declines. In Africa and Asia, fertility also appears to increase sharply in this age bracket, but also remains high in the 25–29 year age group before declining. ASFRs in the 15–19 year age bracket are higher in Latin America than in Asia, and ASFR in Latin America in the 25–29 year age bracket is lower (132) than the corresponding peak in Asia (171). ASFRs in rural areas appear to be higher in all age brackets than in urban areas, and ASFR appears to peak at a younger age in rural areas (20–24 years) than in urban areas (25–29 years; figure 6B). In low-income areas, ASFRs are consistently higher than in lower-middle-income and upper-middle-income areas, and there is a wide overlap in the distribution of ASFRs in lower-middle-income and upper-middle-income countries, suggesting a non-significant difference in ASFRs between these two groups (figure 6C). ASFR in all age brackets is higher in areas where TFR is 4·0 children or more per woman, compared with areas with TFRs of less than 4·0 (figure 6D).

## Discussion

The UN has highlighted the importance of studying the relationship between population, development, and geography to support policy makers in all countries to work to achieve the goals outlined in the 2030 Agenda for Sustainable Development.^32^ The results of this study show the importance of looking at spatial aspects of fertility and spatial autocorrelation of demographic indicators and fertility change.^33^ Moreover, spatial analysis of demographic factors can reveal patterns that could otherwise be overlooked.^16^ The results support previous studies, in which, in India, for example, the identification of spatial clustering of demographic phenomena led to the observation of patterns, providing a better understanding of the nature of demographic differentials in each region.^34^

This study is important in its subnational approach, which enabled us to capture heterogeneities within regions and countries. We found substantial within-country variation in the distribution of TFRs and teenage fertility rates, highlighting the need for geographically tailored programmes and strategies in areas with high fertility and low coverage of family planning services and low proportions of women with secondary or higher education. Fertility rates varied from below replacement in large parts of India, Colombia, and Armenia, where TFRs were as low as 1·1 in some areas, to areas of sub-Saharan Africa and Afghanistan, where TFRs were 8 or higher. The implication of these findings on global population growth are important, especially when considering the population size and urban development of India, which is projected to overtake China as the world's most populous country in 2027.^35^ Areas with below-replacement fertility rates are likely to present different policy and global health challenges than areas with relatively high fertility rates and high unmet need for contraception, because they are at different stages towards the achievement of the SDGs.

Sub-Saharan Africa was found to have the highest concentration of high fertility rates and the highest ASFR across all age groups. As a populous region of 1·1 billion people, these findings have important implications for maternal and child health, the environment, and the economy of these countries.^2^ Sub-Saharan Africa was also found to be characterised by low use of contraception, the highest rates of unmet needs for family planning, and a low percentage of women with at least secondary education, suggesting that social interventions should be focused on areas such as these that are at most disadvantage. The role of family planning programmes on fertility reduction in sub-Saharan Africa has been widely discussed in relation to their geographical distribution, highlighting geographical heterogeneities.^36^, ^37^ In this study, we found that, in all three regions, high-fertility clusters can cross geographical areas that are spatially close but belong to different countries, suggesting that high fertility can remain high irrespective of country-specific family planning programmes. Previous studies have found that family planning interventions implemented through mass media, community discussion, and advocacy from religious leaders worked particularly well in sub-Saharan Africa. Findings from the current study support the importance of women's education in family planning, and show how background characteristics that cluster geographically and often across country borders, such as religion or language group, play a role in making programmes effective.^38^

High prevalence of unmet needs for family planning appear to correspond to high-fertility clusters across all three regions, irrespective of the rate of contraception use. This finding has important implications for family planning and its role in both unwanted births and wanted births; the impact of family planning goes beyond providing access to contraceptive methods and services.^37^ Finally, we found that, within their respective average spatial resolution groups, countries with the highest TFRs do not necessarily have the highest teenage fertility rates, and vice versa, suggesting that patterns of childbearing ages, behaviour, and social norms differ across areas. Understanding these patterns can have implications on policies aimed at reducing teenage pregnancies.

A limitation of the present study is the lack of fully comparable data for some countries. Data sources used in this study come from surveys done over a range of different years (2010–16), and inconsistencies in survey data might affect comparability, especially in areas where fertility rates are changing rapidly. Only a subsample of low-income and middle-income countries have been used in this study, due to the low availability of data from the DHS and the MICS surveys. This limitation could have influenced the clustering; high-fertility and low-fertility clusters are defined as relative to all areas included in the analysis. Furthermore, it was necessary to perform the cluster analysis by region to maintain comparability within region and to be able to identify cross-country hot spots and cold spots, but this approach meant that high-fertility clusters and low-fertility clusters have different magnitudes in each region. Including more data as they become available could affect the cluster analysis and will be a focus of ongoing work. Some limitations exist in the survey data that were used to construct the indicators. Data quality issues such as recall bias, misreporting, and omissions might arise when using fertility data based on birth history data, although a study done in 69 countries using 182 DHS surveys has shown that DHS fertility estimates are of good or acceptable quality in the majority of surveys.^39^ Data were aggregated at subnational level and not at individual level, and so it was not appropriate to look at the correlation of fertility with background characteristics and determinants of fertility, or to assess the effect of programmes, on the basis of data available. In addition, aggregation at DHS and MICS subnational level is likely to obscure some of the finer-scale variations in rates. Finally, due to the modifiable areal unit problem, which recognises that there are different ways of drawing geographical boundaries to summarise data and that they are often demarcated artificially, resulting in different patterns in the data measured,^40^ findings from this study can only be interpreted within the specifically defined areas, and different levels of aggregation could result in different findings.

Future work will aim to broaden the scope of the analyses by expanding the work to global scales, examining multitemporal trends, and integrating other sources of data, including high resolution birth maps and pregnancies for Africa, Latin America, and the Caribbean developed by the WorldPop project,^41^ to construct a more complete picture of overall fertility behaviours.

## Data sharing

The input data used to produce this work are freely available after approval of registration and with a signed data access agreement on the websites of the data providers (ie, DHS and MICS). The outputs produced in this paper can be made available upon request to the corresponding author.

## Declaration of interests

We declare no competing interests.
