# Supplementary material for: Geographical distribution of fertility rates in 70 low-income, lower-middle-income, and upper-middle-income countries, 2010–16: a subnational analysis of cross-sectional surveys
Source: Lancet Glob Health. 2021 May 18;9(6):e802–12. doi: 10.1016/S2214-109X(21)00082-6 (PMC8149299; doi:10.1016/S2214-109X(21)00082-6)
Supplement: Supplementary appendix [file mmc1.pdf]

THE LANCET
Global Health

Supplementary appendix

This appendix formed part of the original submission and has been peer reviewed. We post it as supplied by the authors.

Supplement to: Pezzulo C, Nilsen K, Carioli A, et al. Geographical distribution of fertility rates in 70 low-income, lower-middle-income, and upper-middle-income countries, 2010–16: a subnational analysis of cross-sectional surveys. *Lancet Glob Health* 2021;
9: e802–12.

Appendix

Geographical distribution of fertility rates in 70 low-income, lower-middle-income, and upper-middle-income countries, 2010–16: a subnational analysis of cross-sectional surveys

Carla Pezzulo[1], Kristine Nilsen[1], Alessandra Carioli[1], Natalia Tejedor-Garavito[1], Sophie E Hanspal[1], Theodor Hilber[2], William H M James[1,3], Corrine W Ruktanonchai[1,4], Victor Alegana[5,6], Alessandro Sorichetta[1], Adelle S Wigley[1], Graeme M Hornby[1,7], Zoe Matthews[8], Andrew J Tatem[1]

1. WorldPop, Geography and Environmental Science, University of Southampton, Highfield Campus, Southampton SO17 1BJ, UK
2. Freie Universität Berlin, Department of Earth Sciences, Centre for Development Research (ZELF), Germany
3. School of Geography and Leeds Institute for Data Analytics, University of Leeds, Leeds LS2 9JT, UK
4. Department of Population Health Sciences, Virginia Tech, Blacksburg, VA, USA
5. Population Health Unit, Kenya Medical Research Institute - Wellcome Trust Research Programme, P.O. Box 43640-00100, Nairobi, Kenya
6. Centre for Tropical Medicine & Global Health, Nuffield Department of Medicine, University of Oxford, John Radcliffe Hospital, Headington, Oxford, OX3 9DU, UK
7. GeoData, University of Southampton, Highfield Campus, Southampton SO17 1BJ, UK
8. Division of Social Statistics and Demography and Centre for Global Health, Population, Poverty and Policy, University of Southampton, Southampton SO17 1BJ, UK

SI.1 Data

Sampling design. A stratified two-stages probability sampling design is usually used by the DHS Program to design nationally representative surveys in most of the low and middle income countries, where strata are obtained by combining states (or provinces) with urban and rural areas. In the first stage of selection, primary sampling units (typically census enumeration areas) are selected with probability proportional to size in each stratum. In the second stage, a listing of households usually serves as a sampling frame, and a fixed number of households in each cluster are selected using equal probability systematic sampling in the selected sampling unit. Due to the selection probability method used in sample design, sampling weights were used when undertaking any analysis. The use of sampling weights also corrects for the under- or over-sampling of different strata during sample selection. Due to their design, DHS surveys ensure national, urban and rural and region (or province) representativeness (1).

Table SI.1.1 Summary statistics by country. Average TFRs and teenage fertility rates and standard deviations (SD) for the 70 countries.

COUNTRY	Total fertility Rate average	Total fertility rate SD	Teenage fertility rate average	Teenage fertility rate SD	Macro Region	Survey Year	Country Income Level
AFGHANISTAN	5·41	1·29	80·26	42·9	Asia	2015	Low
ALGERIA	2·77	0·44	10·91	5·42	Africa	2013	Upper-middle
ANGOLA	6·91	1·09	198·89	49·34	Africa	2015-16	Lower-middle
ARMENIA	1·79	0·42	27·82	18·49	Asia	2016	Lower-middle
BANGLADESH	2·27	0·34	112·86	12·16	Asia	2014	Lower-middle
BENIN	5·01	0·59	101·17	33	Africa	2011-12	Low
BURKINA FASO	5·62	0·77	140·46	26·93	Africa	2014	Low
BURUNDI	5·84	0·94	81·4	19·48	Africa	2012	Low
CAMBODIA	2·82	0·43	61·58	26·41	Asia	2014	Lower-middle

CAMEROON	4·77	1·01	122	51·84	Africa	2014	Lower-middle
CHAD	6·3	0·77	176·33	45·79	Africa	2014-15	Low
COLOMBIA	2·27	0·67	93·06	37·45	Latin America	2015	Upper-middle
COMOROS	4·57	0·93	85·67	39·07	Africa	2012	Low
CONGO	5·97	0·81	188·58	43·31	Africa	2011-12	Lower-middle
CONGO DEMOCRATIC REPUBLIC	6·89	1·02	152·38	30·77	Africa	2013-14	Low
COTE IVOIRE	5·4	1·01	141	37·42	Africa	2013	Lower-middle
DOMINICAN REPUBLIC	2·63	0·34	99·68	20·38	Latin America	2014	Upper-middle
EGYPT	3·55	0·52	56·44	18·19	Africa	2014	Lower-middle
EL SALVADOR	2·41	0·33	76·87	14·96	Latin America	2014	Lower-middle
ETHIOPIA	4·35	1·41	86·64	38·29	Africa	2016	Low
GABON	5·06	0·88	154·1	35·41	Africa	2012	Upper-middle
GAMBIA	5·85	1·29	95·38	39·3	Africa	2013	Low
GHANA	4·3	0·97	74·3	25·68	Africa	2016	Lower-middle
GUATEMALA	3·17	0·52	93·05	22·96	Latin America	2014-15	Lower-middle
GUINEA	5·25	0·93	152·5	33·9	Africa	2012	Low
GUINNEA BISSAU	5·45	1·05	118·99	32·22	Africa	2014	Low
GUYANA	3·65	1·56	106·59	60·69	Latin America	2014	Upper-middle
HAITI	3·87	0·7	67·82	14·19	Latin America	2012	Low
HONDURAS	3·15	0·51	107·5	25·73	Latin America	2011-12	Lower-middle
INDIA	2·04	0·45	43·2	22·11	Asia	2015	Lower-middle
INDONESIA	2·78	0·4	56·06	21·79	Asia	2012	Lower-middle
IRAQ	4·68	0·73	83·63	24·53	Asia	2011	Upper-middle
JORDAN	3·76	0·31	21·75	8·68	Asia	2012	Lower-middle
KENYA	4·04	0·78	101·8	21·81	Africa	2015	Lower-middle
KYRGYZ REPUBLIC	4·14	0·69	66·24	33·11	Asia	2014	Lower-middle
LAO PDR	3·34	0·67	104·19	29·12	Asia	2011-2012	Lower-middle
LESOTHO	3·47	0·59	99·4	20·08	Africa	2014	Lower-middle
LIBERIA	5·67	0·97	191·33	39·91	Africa	2013	Low
MADAGASCAR	4·48	0·88	155	26·99	Africa	2016	Low
MALAWI	4·68	0·72	141·58	24·97	Africa	2015-16	Low
MALI	6·32	0·94	175·83	38·5	Africa	2015	Low
MAURITANIA	5·11	0·68	94·2	21·91	Africa	2011	Lower-middle
MONGOLIA	3·26	0·25	51·85	26·15	Asia	2014	Lower-middle
MOZAMBIQUE	5·7	1·25	166·82	35·92	Africa	2011	Low
MYANMAR	2·65	0·83	39·67	12·79	Africa	2015-16	Lower-middle
NAMIBIA	3·92	0·82	98·54	38·05	Africa	2013	Upper-middle
NEPAL	2·34	0·13	87·6	10·95	Asia	2016	Low
NIGER	7·12	1·21	193·38	56·54	Africa	2012	Low
NIGERIA	4·79	0·84	125·89	67·97	Africa	2015	Lower-middle
PAKISTAN	3·63	0·51	40·67	14·18	Asia	2012-13	Lower-middle
PALESTINE	4·13	0·56	50·82	22·15	Asia	2014	Lower-middle
PERU	2·81	0·51	78·17	29·97	Latin America	2010-2012	Upper-middle
PHILIPPINES	3·29	0·52	59·35	10·94	Asia	2013	Lower-middle

RWANDA	4·1	0·48	44·2	14·34	Africa	2014-15	Low
SAO TOME	4·55	0·22	94·14	7	Africa	2014	Lower-middle
SENEGAL	5·45	1·12	96·81	41·99	Africa	2016	Low
SIERRA LEONE	4·1	0·54	138	26·06	Africa	2016	Low
SOUTH SUDAN	7·52	0·57	163·17	28·84	Africa	2010	Low
SUDAN	5·33	1·15	92·88	28·05	Africa	2014	Lower-middle
SWAZILAND	3·35	0·26	90·32	13·78	Africa	2014	Lower-middle
TAJIKISTAN	3·62	0·41	47·8	16·89	Africa	2012	Lower-middle
TANZANIA	5·43	1·29	127·37	51·15	Africa	2015-16	Low
TOGO	5·05	0·83	91·17	24·31	Africa	2013-14	Low
TUNISIA	2·12	0·17	3·79	3·16	Africa	2012	Lower-middle
TURKMENISTAN	3·1	0·4	28·74	13·2	Asia	2016	Upper-middle
UGANDA	5·78	1	140	31·41	Africa	2014	Low
VIETNAM	2·5	0·31	50·86	31·46	Asia	2014	Lower-middle
YEMEN	4·6	0·97	67·29	17·21	Asia	2013	Lower-middle
ZAMBIA	5·67	1·01	152·9	30·18	Africa	2013-14	Lower-middle
ZIMBABWE	4	0·75	116	33·88	Africa	2015	Low

Table SI.1.2 Definition of the fertility determinants used in the study, extracted from the Measure DHS datasets.

**Indicator name**	**Description**
Use of any contraceptive method	Percentage of currently married or in union women currently using any method of contraception.
Use of modern methods	Percentage of currently married or in union women currently using any modern method of contraception.
Unmet need for family planning	Percentage of women who do not want to become pregnant but are not using contraception. For detailed discussion on the construction of the indicator see the measure DHS page at: https://dhsprogram.com/topics/unmet-need.cfm
Women's level of education	Percentage of women with secondary or higher education.

SI.2 Description of TFR and teenage fertility rates data for the countries in study.

Figure SI.2.1. Distribution of subnational teenage fertility rates (age specific fertility rates for the age group 15-19 years) for 70 countries in Africa, Asia and Latin America collected between 2010 and 2016. Coloring scheme ranges from yellow (low rates) to brown (high rates). The rates refer to the number of births occurring during the three years preceding the survey per 1,000 women of reproductive age. *No data indicate low, lower-middle and upper-middle income countries where no data between 2010 and 2016 were available. In dark grey are indicated upper income countries.

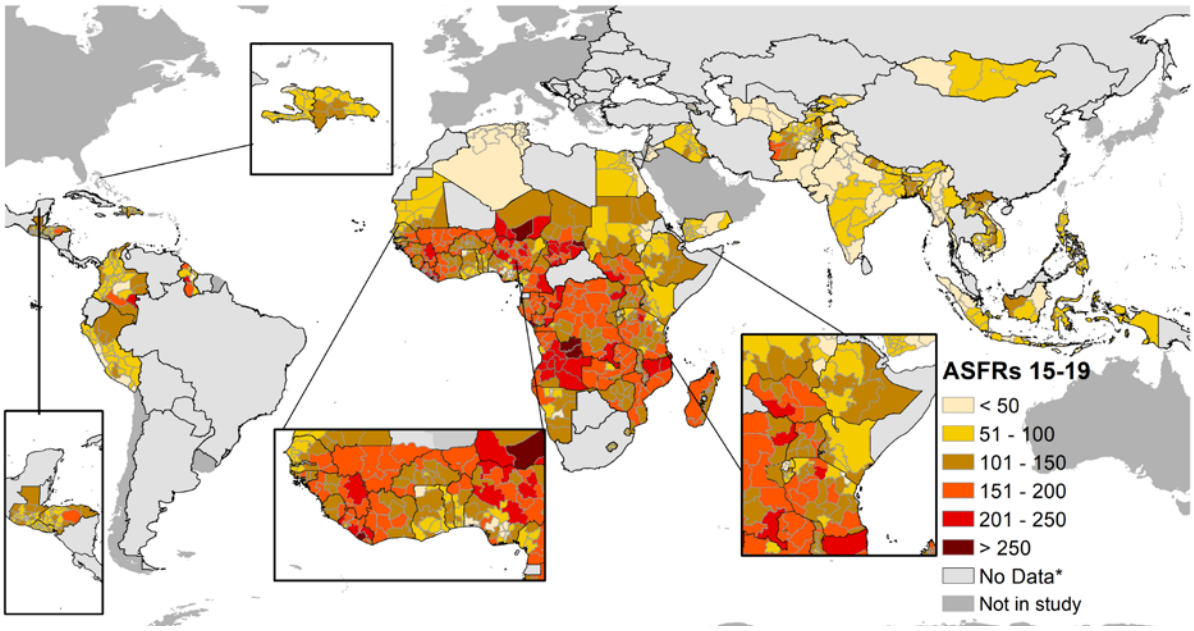

Highest rates of teenage fertility (ASFR15-19 equal to 200 or higher) were found in areas of Central and Wester Africa, including Angola, areas of Mozambique, Chad and Cameroon, Nigeria and Niger, Liberia and Mali, and a few areas of DRC and South Sudan. Only few areas of Colombia and Guyana showed high teenage fertility rates.

Figures SI.2.1.1 a and b show the 95% confidence interval width for the (a) TFR and (b) ASFR15-19 rate values for the subnational areas in Africa, Latin America and Asia.

Figure SI.2.1.1 Maps of 95% confidence interval width for the (a) TFR and (b) ASFR15-19 rate values for the subnational areas in AF, LA and AF.

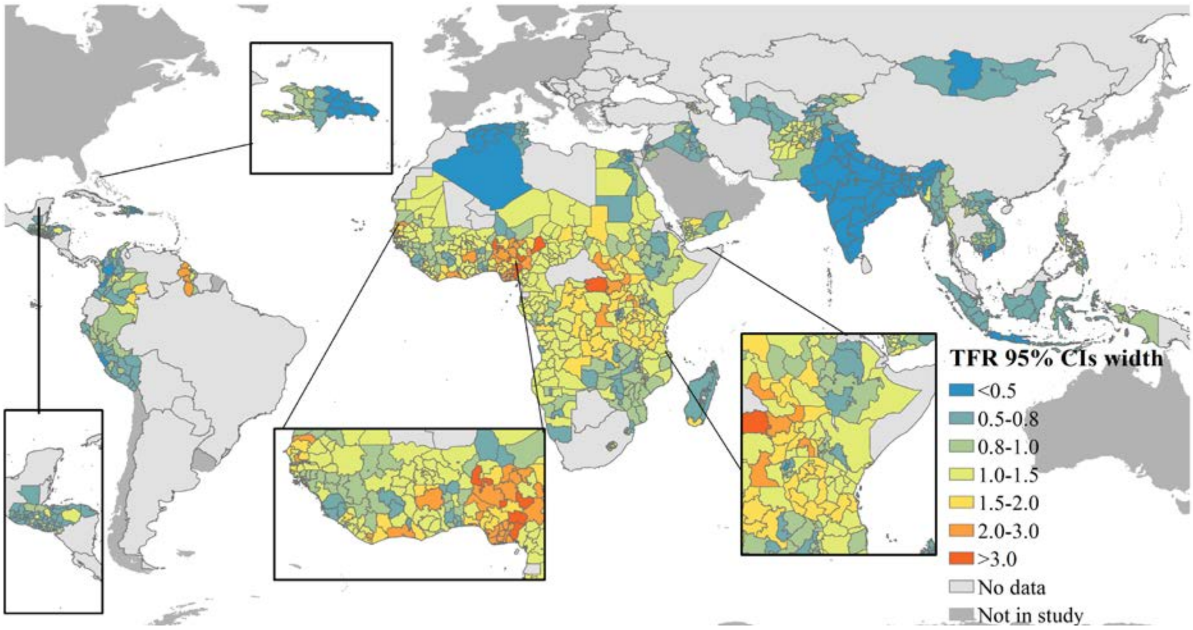

(a)

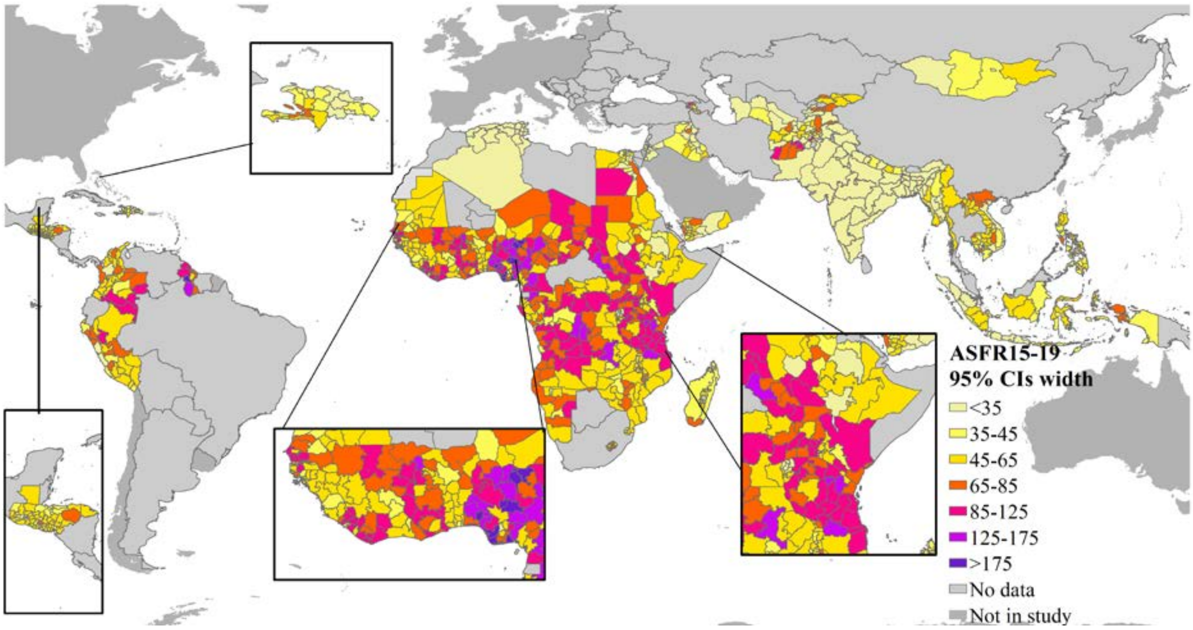

(b)

Table SI.2 Summary statistics high and low fertility clusters in (a) Latin America, (b) Africa and (c) Asia

	Mean TFR	Standard Deviation	Median TFR	Min value	Max value
**Latin America**					
No significant clustering	2.97	0.807	2.9	1.6	6.5
High fertility cluster	3.20	0.731	3.4	2.3	4.2
Low fertility cluster	2.06	0.512	2.1	1.3	3.4
Total	2.88	0.826	2.8	1.3	6.5
**Africa**					
No significant clustering	5.11	1.186	5.1	1.8	8.5
High fertility cluster	7.09	0.787	7.1	5.1	8.6
Low fertility cluster	3.05	0.684	3.1	1.9	4.4
Total	5.18	1.414	5.2	1.8	8.6
**Asia**					
No significant clustering	3.37	1.071	3.2	1.2	6.6
High fertility cluster	5.24	1.403	4.9	2.8	8.9
Low fertility cluster	2.03	0.633	1.9	1.1	4.6
Total	3.42	1.320	3.2	1.1	8.9

Figure SI.2.1.2 Distribution of Total Fertility Rates by high and low fertility clusters in (a) Latin America, (b) Africa and (c) Asia. The box shows the median (50th percentile, or middle value), lower quartile (25th percentile) and upper quartile (75th percentile), while the wiskers show minimum and maximum values. The dots outside the wiskers represent outliers.

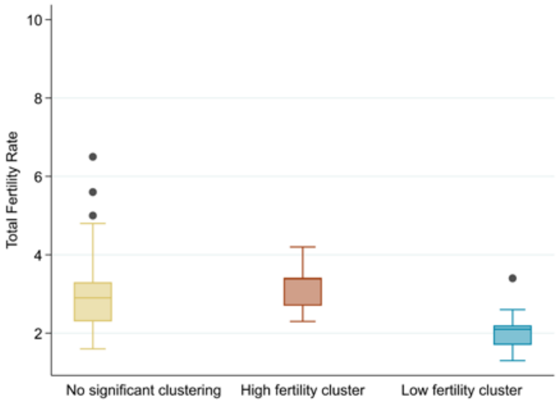

(a)

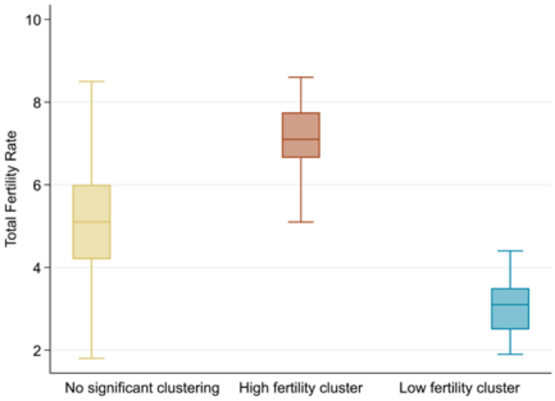

(b)

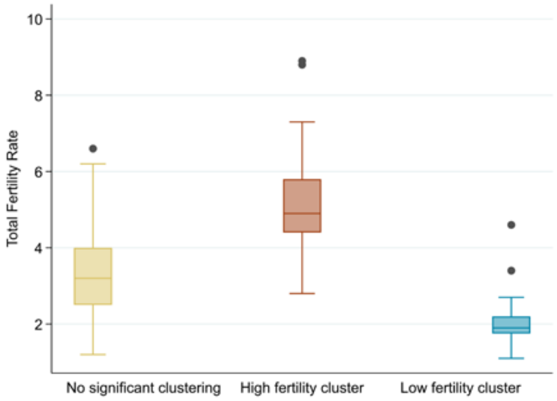

(c)

As a measure of within country heterogeneity we show the subnational variation of TFRs and teenage fertility rates among all countries included in the analysis, by average spatial resolution group and highlight differences between subnational values and national estimates (Figures SI.2.2-SI.2.3). The average spatial resolution measures the effective resolution of administrative units in kilometers (i.e., the cell size of administrative units if all units were square of equal size), and is calculated as the square root of the land area divided by the number of administrative units (2, 3). The average spatial resolution was calculated, and countries ranked based on their average spatial resolution and grouped by using the median to allow for comparability between countries with different numbers of administrative units and to be able to observe within country variations in rates (see Figure SI.2.4).

Figures SI.2.2 highlight the within country variability in the distribution of the teenage fertility rates by the two average spatial resolution groupings, which would be masked when only looking at country-level estimates. Senegal, Guyana, Gambia and Uganda in ASR group one and Namibia, Cameroon, Nigeria, Tanzania, Chad, Angola, Congo and Niger in ASR group two present the highest heterogeneities in teenage fertility rates. Interestingly, despite average spatial resolution group one has low ASFRs relative to average spatial resolution group two, some countries in average spatial resolution group one present quite a wide range of fertility variation within. When looking at TFRs (Fig. SI.2.3), in many cases the same countries showing high within country variation in teenage fertility rates, also present high variations in TFRs. Some exceptions exist. Congo and Chad present high within country variation in teenage fertility rates but low in TFRs. On the contrary, Yemen, Gambia, DRC and Mozambique present high within country variation in TFRs and low variation in teenage fertility rates, suggesting different onset-timing of childbearing and similar within country behaviours among adolescents. In every average spatial resolution group, patterns of teenage fertility rates (following the ordering from low rates to high rates) do not reflect patterns of TFRs (from low rates to high rates). Within their respective average spatial resolution group, countries with the highest TFRs do not necessarily have the highest teenage fertility rates, and vice versa: e.g. Burundi (average spatial resolution group one) presents the highest TFR, but not the highest teenage fertility rates among his average spatial resolution group, meaning that fertility is still highly influenced by later age births. Conversely, Bangladesh shows low TFRs but high teenage fertility rates. Colombia and Nepal (average spatial resolution group two) have low TFRs but relatively high teenage fertility rates, compared to other countries in the same average spatial resolution group. This indicates that patterns of fertility and the correlation between teenage fertility rates and TFRs can vary widely. In countries where teenage fertility rates are low and TFR is high, childbearing age is delayed towards later ages, perhaps due to social norms such as having births within marriage or having the first child after completion of a certain school grade. Figure SI.2.5 confirms that some subnational areas show high TFRs but low teenage fertility rates, e.g. Anambra in Nigeria and Paktika, Laghman and Zabdul in Afghanistan as well as areas in Senegal and Tanzania, indicating that childbearing is shifted towards later ages and that only few children are born from adolescents. On the contrary, other subnational areas have high teenage fertility rates and low TFRs (e.g. Caqueta and Guainia in Colombia; Rajshahi and Rangpur in Bangladesh and Luangnamtha in Lao PDR), meaning that fertility is low among older ages, which may suggest that the ideal number of total children has been reached and that perhaps family planning is being used. In many of these cases, this may indicate that family planning policies have an impact on reducing the numbers of births over later ages.

Table SI.2.1 Medians (p50), lower and upper quartiles (p25 and p75) and interquartile ranges (iqr) for key determinants of fertility in high and low fertility clusters in Africa, Asia and Latin America.

	**Any contraceptive method**				
		**p50**	**p25**	**p75**	**iqr**
Africa					
	High fertility cluster	9.65	4.30	15.12	10.82
	Low fertility cluster	58.70	51.60	63.23	11.63
Asia					
	High fertility cluster	21.65	14.40	32.70	18.30
	Low fertility cluster	59.85	53.15	65.40	12.25
Latin America					
	High fertility cluster	42.40	40.80	43.80	3.00
	Low fertility cluster	83.40	80.60	84.50	3.90
	**Modern contraceptive methods**				
		**p50**	**p25**	**p75**	**iqr**
Africa					
	High fertility cluster	4.43	2.59	8.19	5.60
	Low fertility cluster	58.20	54.40	62.04	7.64
Asia					
	High fertility cluster	19.30	13.30	26.90	13.60
	Low fertility cluster	49.95	26.65	57.00	30.35
Latin America					
	High fertility cluster	40.30	38.20	41.70	3.50
	Low fertility cluster	77.00	72.30	79.90	7.60
	**Unmet need for family planning**				
		**p50**	**p25**	**p75**	**iqr**
Africa					
	High fertility cluster	27.64	22.50	32.80	10.30
	Low fertility cluster	10.00	8.10	12.11	4.01
Asia					
	High fertility cluster	25.75	22.80	30.60	7.80
	Low fertility cluster	11.15	7.70	13.70	6.00
Latin America					
	High fertility cluster	31.80	28.00	31.80	3.80
	Low fertility cluster	6.40	4.70	9.30	4.60
	**Secondary and higher education**				
		**p50**	**p25**	**p75**	**iqr**
Africa					

	High fertility cluster	21.10	12.53	32.06	19.53
	Low fertility cluster	62.18	44.15	73.02	28.87
Asia					
	High fertility cluster	8.80	4.50	14.40	9.90
	Low fertility cluster	69.60	54.65	99.50	44.85
Latin America					
	High fertility cluster	72.10	48.20	72.70	24.50
	Low fertility cluster	80.80	79.50	81.90	2.40

Figure SI.2.2 Differences between national and subnational teenage fertility rates (ASFRs 15-19) by average spatial resolution group. Panel 2.2(a) shows ASFRs 15-19 for countries in average spatial resolution group 1 and Panel 2.2(b) shows ASFRs 15-19 for countries in average spatial resolution group 2. Teenage fertility rates as measured as births per 1,000 women of age 15 to 19. Country box plots are constructed by using aggregated administrative level rates. For each country, the corresponding yearly national estimate (red dots) is shown in comparison with the year of the subnational data. The blue line represents the average teenage fertility rate in 2017 for high-income countries (22 births per 1,000 women of age 15-19). Group one includes countries where average spatial resolution is < 156, group two: >=156. Figure SI.2.4 maps countries in the study area by average spatial resolution groups.

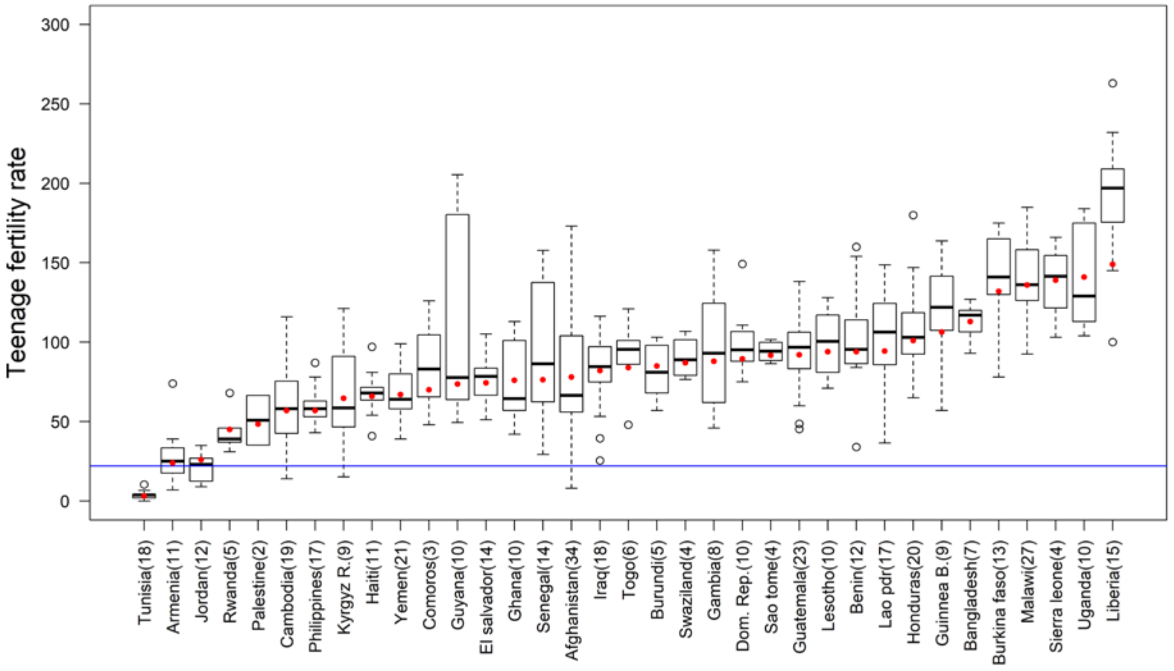

a)

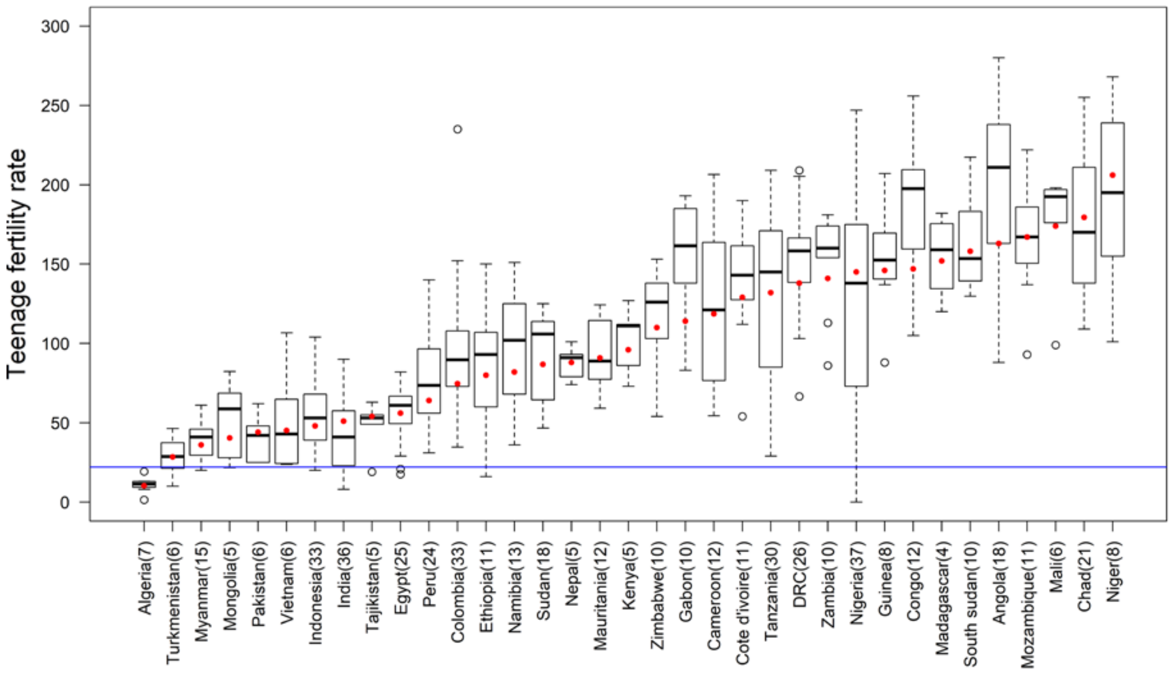

b)

Figures SI.2.3 Differences between national and total fertility rates by average spatial resolution group.

Panel SI.2.3(a) shows total fertility rates for countries in average spatial resolution group one and Panel SI.2.3(b) shows total fertility rates for countries in average spatial resolution group two. Country box plots are constructed by using aggregated administrative level rates. For each country, the corresponding yearly national estimate (red dots) is shown in comparison with the year of our data. The blue line represents the average TFR in 2017 for high-income countries (1·7).

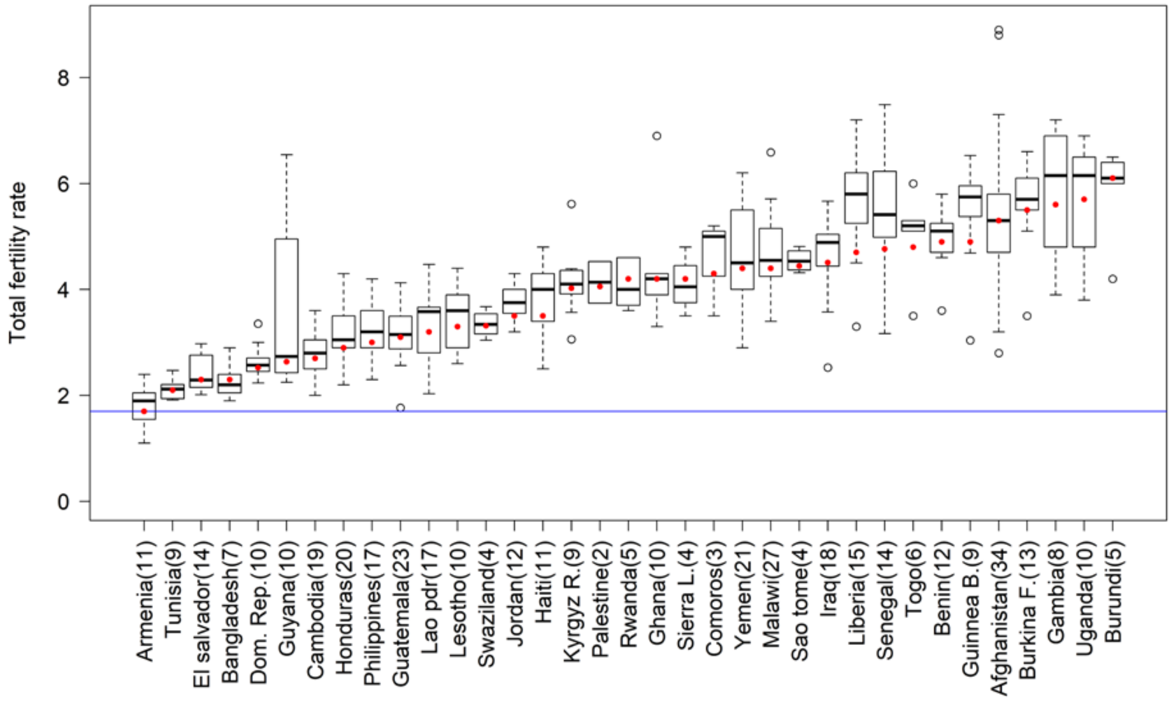

a)

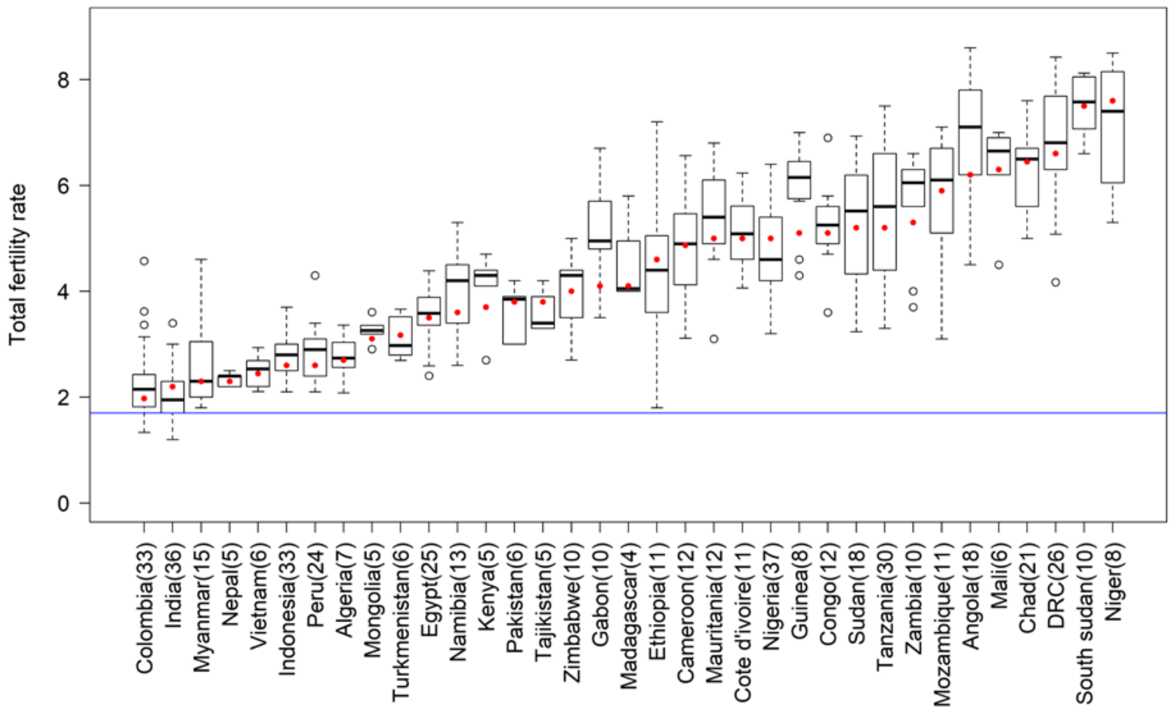

b)

Figure SI.2.4 Countries by average spatial resolution group. The map shows the countries by average spatial resolution groups. To allow comparability between countries with different number of administrative units, the average spatial resolution for each country was calculated, and countries were ranked based on their average spatial resolution and grouped by using the median (156). The average spatial resolution measures the effective resolution of administrative units in kilometers, and is calculated as the square root of the land area divided by the number of administrative units (2, 3). Group one includes countries where average spatial resolution is < 156 and group two: >=156.

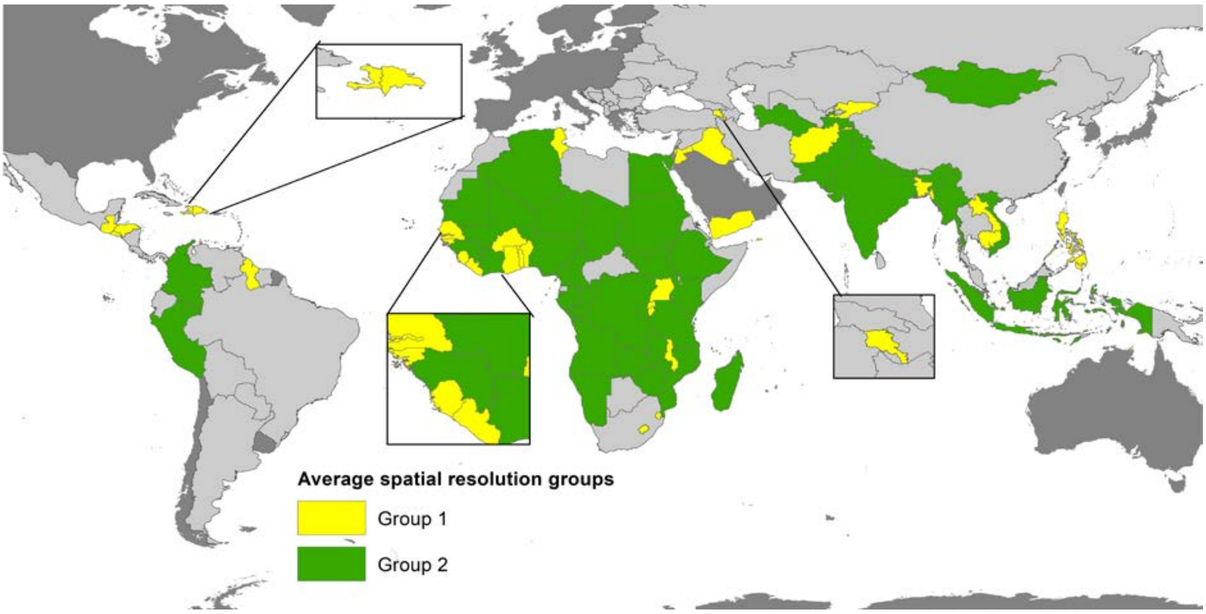

Figure SI.2.5. Subnational teenage fertility rates by total fertility rates. Subnational areas are color-coded by continent = Asia: green; Latin America: blue; Africa: red. This figure allows us to examine whether there is any association between the TFRs and the teenage fertility rates (ASFRs 15-19). Countries with an earlier onset of fertility have a higher total level of fertility.

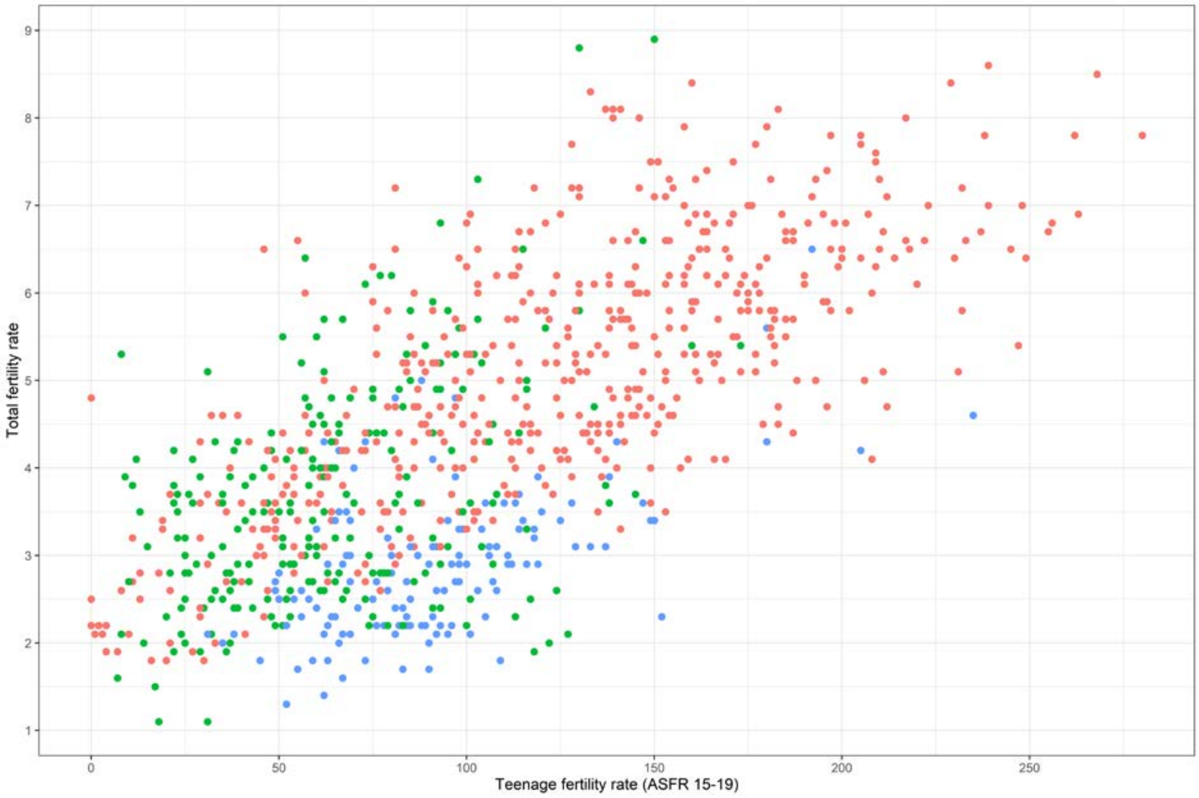

Table SI.3 Measures of spatial autocorrelation measured by Global Moran's I (univariate) run in GeoDa software with a pseudo p-value: <0·001 with 999 permutations for the four determinants of fertility.

	Latin America	Africa	Asia
Total fertility rate	0·41 (z-score: 7·6)	0·62 (z-score: 22·1)	0·72 (z-score:16·4)
Use of any contraceptive method	0·78 (z-score: 13·3)	0·78 (z-score: 25·9)	0·69 (z-score: 14·9)
Use of modern methods of contraception	0·72 (z-score: 11·9)	0·82 (z-score: 27·1)	0·68 (z-score: 14·6)
Unmet need for family planning	0·73 (z-score: 12·6)	0·63 (z-score: 20·9)	0·65 (z-score: 14·2)
Secondary or higher education	0·76 (z-score: 13·1)	0·69 (z-score: 22·5)	0·80 (z-score: 17·9)

Figure SI.4 Clusters of areas with high and low prevalence of determinants of TFRs, including a) use of any contraceptive methods, b) use of modern methods, c) unmet needs for family planning and d) education (secondary or higher), in (i) Latin America, (ii) Asia. Clusters were calculated using randomization (n=99999 permutations) and a pseudo p-value of <0·01 as the cut off value. Please note that the colour scale shows in red high level clusters for each variable, and in light blue low level clusters for each variable, irrespective of the sign of the variable: red may indicate high risk (as in the case of unmet needs for family planning) or high protection (as in the case of use of contraception and percentage of women with secondary or higher education.

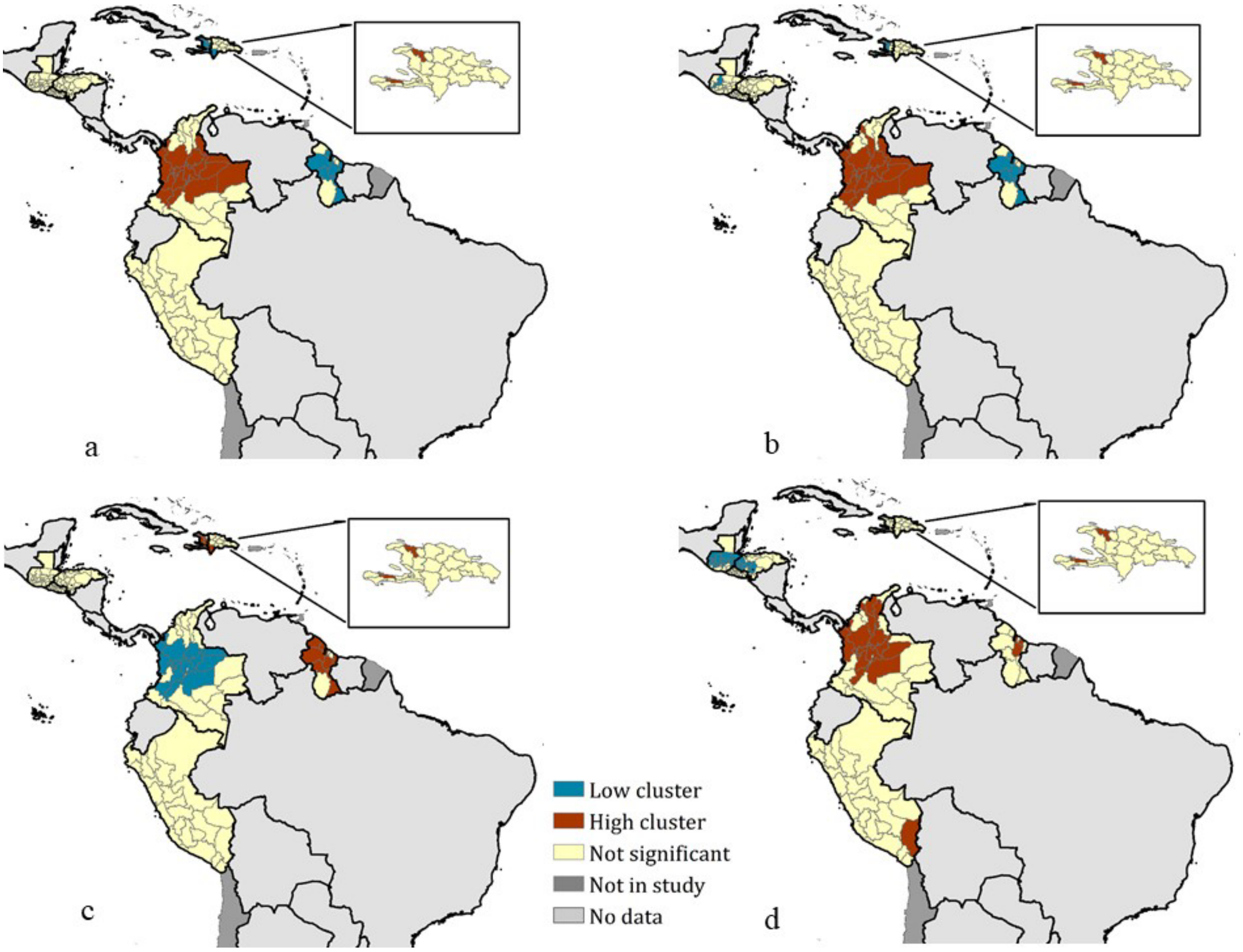
a
b
c
d
Low cluster
High cluster
Not significant
Not in study
No data

(i)

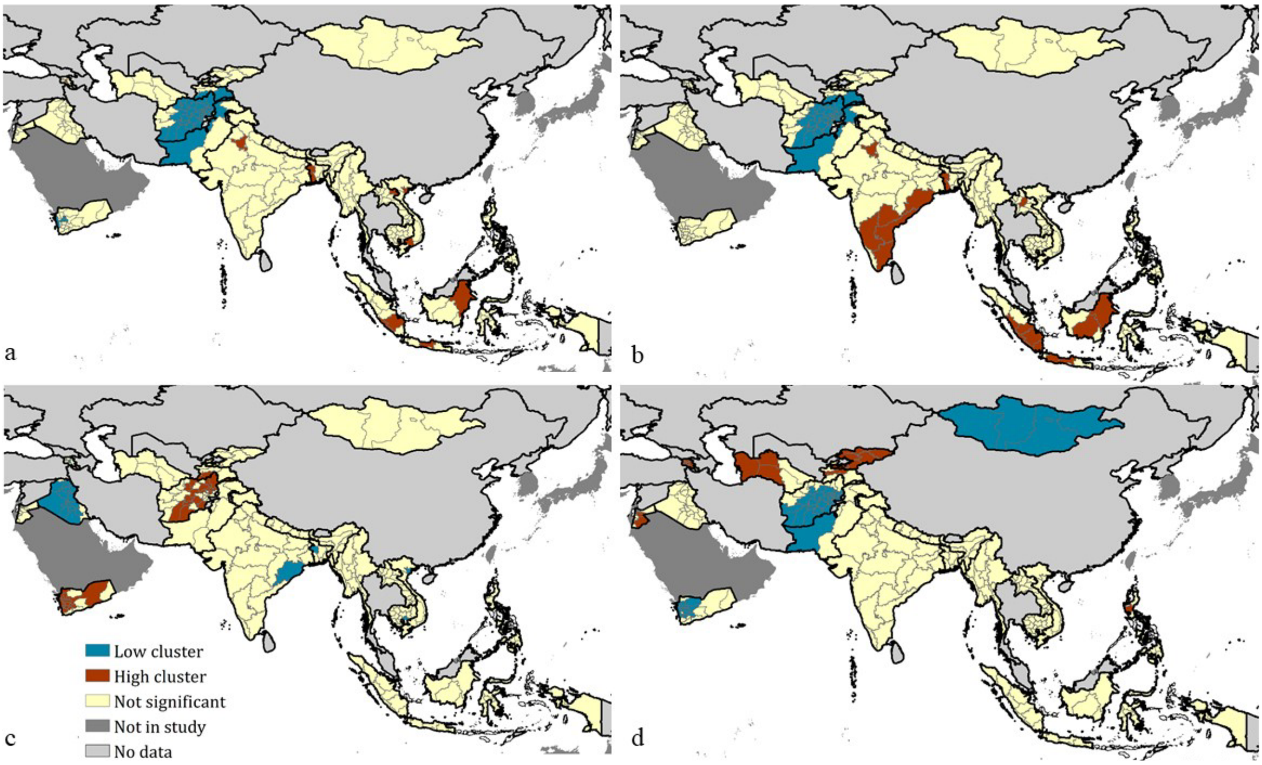
a
b
c
d
Low cluster
High cluster
Not significant
Not in study
No data

(ii)

1. ICF International. Demographic and Health Survey Sampling Household Listing Manual. ICF International.; 2012.
2. Balk D, Yetman G. The Global Distribution of Population: Evaluating the gains in resolution refinement. London: Center for International Earth Science Information Network (CIESIN); 2004.
3. Linard C, Alegana VA, Noor AM, Snow RW, Tatem AJ. A high resolution spatial population database of Somalia for disease risk mapping. International Journal of Health Geographics. 2010;9(1):45.
